# Understanding the Lived Experience of North American Dental Patients With a Single-Tooth Implant in the Upper Front Region of the Mouth: Protocol for a Qualitative Study

**DOI:** 10.2196/25767

**Published:** 2021-06-18

**Authors:** Kelvin Ian Afrashtehfar, Stephen Ross Bryant

**Affiliations:** 1 Division of Restorative Dental Sciences Clinical Sciences Department Ajman University Ajman City United Arab Emirates; 2 Department of Reconstructive Dentistry and Gerodontology School of Dental Medicine University of Bern Berne Switzerland; 3 Centre of Medical and Bio-allied Health Sciences Research Ajman University Dubai United Arab Emirates; 4 Department of Oral Surgery and Stomatology Faculty of Medicine University of Bern Berne Switzerland; 5 Department of Oral Health Sciences Faculty of Dentistry University of British Columbia Vancouver, BC Canada; 6 Division of Prosthodontics and Dental Geriatrics Faculty of Dentistry University of British Columbia Vancouver, BC Canada

**Keywords:** esthetic dentistry, esthetics, implant dentistry, patient perception, patient-reported outcome measures, personal satisfaction, phenomenology, single-tooth dental implants, single-unit implant-supported restoration

## Abstract

**Background:**

Assessment of the subjective experiences of individuals with maxillary anterior (ie, the upper front region of the mouth) single-tooth implants is limited mainly to quantitative measurements of satisfaction with appearance. Interestingly, there is unexplained variability in the relationship between satisfaction and appearance.

**Objective:**

This qualitative study protocol aims to explore and better understand the satisfaction with appearance and function in a Canadian population with maxillary anterior single-tooth implants treated at a postgraduate university clinic. Thus, we aim to obtain diversity among participants relating to the identification of esthetically pleasing and displeasing cases from a clinician perspective.

**Methods:**

A qualitative research design using interpretative phenomenology analysis (IPA) will provide an adaptable inductive research approach. The participants will be recruited, and consent documents, photographs, digital intraoral scans, and self-administered questionnaire responses will be obtained from them. The transcribed verbatim data from audio-recorded, in-depth, semistructured, one-to-one interviews of the participants will be managed, coded, and analyzed thematically with computer-assisted qualitative data analysis software. The IPA will consider the COnsolidated criteria for REporting Qualitative (COREQ) guidelines when applicable.

**Results:**

For the qualitative interview, we plan to include at least eight patients to conduct up to 1.5 hours of open-ended interviews with each participant aided by an interview guide. Ethical approval was granted by the University of British Columbia Behavioral Research Ethics Board (H19-00107) in May 2019. Two American dental foundations funded this study.

**Conclusions:**

The analysis in this study will elucidate the aspects (including their value) that influence participant satisfaction at different dental implant treatment stages. This will be the first qualitative study on this group of the population to explore and obtain a better understanding of their satisfaction with appearance and function, as well as any other patient-reported outcome measures that could be identified.

**International Registered Report Identifier (IRRID):**

DERR1-10.2196/25767

## Introduction

### Background

A single missing maxillary tooth in the esthetic zone (ie, tooth sites that are visible in the smile) is increasingly managed with a dental implant, especially when the adjacent teeth are relatively free from disease or damage. At the moment, the problem is that there is an incomplete understanding of the experiences and perceptions patients have with the treatment outcomes of anterior single-tooth implants. Although the prevalence of tooth loss has been decreasing in recent decades [1], up to one-quarter of adults in Western countries are missing at least one anterior tooth [2,3].

It has been indicated that early reports on patient-reported outcome measures (PROMs) in implant dentistry focused on general patient satisfaction, which may not serve to adequately assess the range of impacts of implants on treatment outcomes as perceived by patients. Thus, researchers recommended adding more detailed questions to provide insight into a broader range of aspects that might affect patient satisfaction with implant prostheses [4-12]. Naturally, patient satisfaction with maxillary anterior single-tooth implants is likely influenced by appearance in addition to a range of outcomes broadly related to function, including maintenance and complication issues, and other factors like body image, patient expectations, and financial restrictions, as well as successfully re-establishing comfortable oral function and stable dental occlusion [13-16]. Understanding patient functional experiences, perhaps most notably involving chewing and speech, will be useful for discussing realistic functional outcomes with patients relative to their expectations [17,18]. Having a more thorough explanation of this from a patient perspective would be useful in further understanding patient satisfaction with maxillary anterior single-tooth implants.

It is noteworthy that qualitative studies concerning patient accounts of their experiences with dental implants are limited [19]. The only qualitative study of patients with single-implant crowns focused on the posterior zone [20]. To our knowledge, no study has explored experiences and perceptions among patients with single implants in the anterior zone. Therefore, the ultimate purpose of this research study is to provide a deeper understanding of the lived experience of Canadian patients who have received a single implant in the anterior zone in a university setting. The results from this study will constitute the first dedicated evidence to address this question qualitatively. The analysis of the findings may provide tools to clinicians for improved understanding and communication with this group of the dental population.

### Objectives of the Study

This study aims to (1) provide a deeper understanding of patient experiences and perceptions with a single-tooth implant in the anterior zone and (2) explore their satisfaction with their perceived outcomes.

### Research Question

The research question is as follows: “What are the experiences and perceptions of patients relating to their satisfaction with a single-tooth implant in the maxillary esthetic region?”

## Methods

### Design

The study’s aim requires a holistic assessment of the phenomenon in question, which suggests using an inductive qualitative method that allows for broad exploration, including matters that may have been overlooked with existing quantitative approaches. The qualitative research design used to address the research question will be adapted from an interpretative phenomenological analysis (IPA) [21-23].

To optimize the opportunity for participants to tell their stories, the researchers need to acknowledge and try to mitigate the effects of the unequal power relationship that regularly exists between researchers and their participants [24]. Semistructured open-ended interviews play an important role here, since they offer the possibility of contradicting researchers’ preconceived categories of understanding [25-30].

van Manen [31] emphasized that the highest value of phenomenological research in the health sciences may not lie so much in its potential for understanding treatment outcomes and stated, “The ultimate aim of a phenomenology of practice is modest: to nurture a measure of thoughtfulness and tact in the practice of our professions and in everyday life.”

Contrary to following a defined set of methods, IPA involves adjusting an approach to thinking; thus, it can easily become challenging [31]. Thus, the researchers will subscribe to van Manen’s recommendation for a dynamic interaction between six research steps that allow flexibility in working intermittently or simultaneously, back and forth between steps, as a form of an “interpretative circle,” depending on the evolving research needs [31,32].

### Planning and Developing the Method Based on van Manen’s Framework

#### Step 1: Turning to the Nature of Lived Experience

This step involves framing a research question. The deep questioning of a research subject will encourage researchers to reflect on their thoughts more profoundly, which initiates the interpretation process. For instance, the research question associated with the phenomenon will constantly be on the researchers’ mind, which will allow for its intentional refinement in the context of this study.

#### Step 2: Investigating Experience as We Live It

An important point to “as we live it” is that the researchers shall abandon preconceived notions on a topic since our experience is full of assumed prejudices. Interpreting a lived experience is both the aim and the source of IPA. Thus, every part of a participant’s life world (ie, the world as immediately experienced, not only the natural world, but also the world of values and human practices) needs to be scrutinized for lived experience material to generate ideas about its essence. Probing questions will facilitate the assessment. For example, “How important is this to you?” and “Does this aspect have any other meaning for you?” are two of several probing questions used to support the interview guide.

#### Step 3: Reflecting on the Essential Themes That Characterize the Phenomenon

In this step, content analysis and the determination of essential themes are accomplished. The analysis is subjected to scrutiny by reflecting on the recognized themes. Additionally, it aims to extract the essential meaning of a phenomenon by asking what constitutes the nature of this lived experience. Thus, this study will try to explore what constitutes the nature of perceptions associated with having a single-tooth implant in the esthetic zone and how these perceptions are shaped by beliefs, values, and needs. A lived experience in an interview context is really the spoken perception of lived experience and even the participant’s interpretation of these perceptions.

#### Step 4: Describing the Phenomenon in the Art of Writing and Rewriting

This is particularly critical in the analytic phase, where writing is integral to the interpretive process rather than simply its final step. Concerning the integral nature of writing, van Manen [32] stated, “To write is to measure our thoughtfulness. Writing separates us from what we know and yet it unites us more clearly with what we know. Writing teaches us what we know, and in what way we know what we know.”

Moreover, writing demands the researchers display the interpretive views on paper and thereby externalize what is inside. In other words, the thoughts and feelings of the participants become perceptible through writing.

#### Step 5: Maintaining a Strong and Oriented Relation to the Phenomenon

In this step, producing appropriate depth and richness in the written text helps researchers remain attuned to the central research question. Not doing so may yield written interpretations with overly superficial speculations or presumptions. Thus, the researchers will try to persist with an intentional focus on reflecting participant experiences related to the research question.

#### Step 6: Balancing the Research Context by Considering the Parts and the Whole

IPA aims to construct text as a comprehensive representation of the phenomenon. In the process, van Manen suggests it is essential that researchers pay attention to each evolving part concerning the whole of one’s study. As such, the results of this study will be interpreted and presented by arranging them as themes and subthemes, relating these to the “whole” relative to the research question.

### Setting

This is a single-center study involving a postgraduate teaching clinic at the University of British Columbia (UBC) Faculty of Dentistry in Vancouver, Canada. The dental specialties of Periodontology/ Periodontics and Prosthodontology/ Prosthodontics are involved in the implant surgery and the implant planning and restoration, respectively.

### Patient and Public Involvement

There is no patient or public participation in the design or discussion of this qualitative study protocol.

### Qualitative Data Collection

To conduct this study, in-depth, semistructured, open-ended interviews using an interview guide will be conducted to collect data. The interviews might be pilot tested.

### Participant Recruitment and Informed Consent

Participants will be recruited from among existing Faculty of Dentistry patients. Postgraduate dental students from the Prosthodontics and Periodontics specialty programs will be contacted and exposed to a standardized study advertisement to identify potential participants among their assigned patients, after which the students will provide the advertisement to potential participants. The students will not share the patients’ names or contact information with the researchers without first obtaining the patients’ permission. Once potential participants identify themselves to the researchers by stating interest in participating in the study, a letter of initial contact will be sent via email, followed by a tentative invitation to participate in the study if their interest is confirmed. Next, an informed consent form will be sent to the participant candidates more than 48 hours in advance of arranging a time to meet for data collection. When each potential participant arrives at the clinic, the informed consent will be reviewed with the aim of having their questions answered and the informed consent signed if agreeable. The ranges of participant demographic characteristics will also be obtained and presented as a group and individually.

### Participant Interviews

The primary data collected for this project will be qualitative participant perceptions gathered through a semistructured, one-to-one, in-depth interview with each participant, using an interview guide based on open-ended questions (Textbox 1 and Multimedia Appendix 1), guided by literature on the subject and the research question [33]. An advantage of face-to-face interviews is that they can often be conducted in a relaxed atmosphere to offer better communication than telephone interviews to develop rapport potentially; however, the cost is higher [34].

**Interview topic guide.**

Part 1: Introductory background questions (icebreaker)

Part 2: Is about your overall satisfaction with the implanted tooth

Part 3: Is about your satisfaction with the appearance (or look) of your implanted tooth

Part 4: Is about satisfaction with your functioning and social experiences relating to your implanted tooth

Part 5: Any other important experiences that affect satisfaction with your implanted tooth, such as complications, maintenance, and financial aspects

Part 6: Surgical aspects of the implant-tooth treatment

The interview guide to be used in this study will contain several central questions in an open format to stimulate the interviewee to dialogue, as well as probing questions to evoke past experiences and to stimulate more reflective thinking. Phenomenological questions seek to reveal perceived meaning in the experiences related to the phenomenon. Therefore, phenomenological description refers to understanding the meaning of a phenomenon. This resulting description and interpretation are not associated primarily with outer knowledge based on generalizing observations and measurable data [31,32]. Moreover, phenomenological questions should not necessarily be seen as complete, in an a priori fashion more typical of deductive investigations, and are more usually ambiguous and unfinished, especially at the outset. However, in the context of health science research, these questions might be more profoundly understood to enable health professionals (eg, dental clinicians) to become more openly sympathetic and sensitive to the difficult situations that patients could face [31].

Additionally, the participants’ identities would remain anonymous and their answers would be independent of their continued opportunity for clinical care. It will be clearly explained to the participants that these interviews would not have any consequence for their future relationship with their dental specialty students or staff.

After establishing contact with each participant and collecting their basic clinical data (as noted), they will be invited individually to a small quiet seminar room near the dental clinic to have a conversation in privacy. Additionally, active listening techniques will be used to encourage participants to elaborate on their experiences but without unnecessarily interrupting them [35]. The researchers will audio record the interview using two small digital recorders only with the participant’s consent. The reason for double recording is in case one recorder malfunctions. The participants will be informed when recording is started and stopped. When the interview concludes, participants will be asked if they could be contacted again if there is a need for clarification. The informed consent states the authorization for audio recording the participants’ interviews. If some participants decide that they do not approve the audio recording of the interview at any stage, they will be excluded from the study.

### Reflective Methods for Data Analysis

As an analytical process, phenomenological reflection aims to understand the central meaning of an experience and of conglomerations of experiences as reflecting the phenomena of interest, which can become a strenuous and challenging assignment [31]. As mentioned earlier, the philosophical basis for phenomenology intentionally avoids following a prescriptive scheme for analysis; thus, “data explication” may be a more accurate term in this context than “data analysis.” Data explication is fundamentally a multilayered progression of defining emerging themes [31,36]. Nonetheless, it is necessary to use the idiographic and hermeneutic philosophical basis of phenomenology in the data explication process [35,37].

#### Thematic Statement Isolation

The analyzed interview transcriptions will be tabulated initially as anonymized analytical themes with the assistance of NVivo version 12 qualitative software (QSR International Pty Ltd) [38,39]. Consistent with an emerging IPA approach, the qualitative themes will be developed from analysis of the transcript data derived from the first three interviews to develop additional avenues for the emerging investigation, which will be adopted through an ongoing iterative modification of the interview guide for subsequent interviews. A word frequency query will be used to verify possible themes at the early stages in the project. The major and minor themes related to the research question will be narratively presented.

Isolation of thematic statements will commence after the interviews are transcribed. van Manen has proposed the following three approaches for isolating themes [32]:

1. The detailed or line-by-line approach: The researchers question the meaning of each sentence related to the phenomenon only after paying close attention to the sentence.
2. The highlighting or selective approach: The researchers question the sentences that appear crucially related to the experience of interest, which are then highlighted after iteratively reviewing the related paragraphs.
3. The holistic reading approach: In reviewing the whole text (or at least a whole section of text), researchers’ questioning of an individual sentence reveals the meaning of the text as a whole, in the context of the phenomenon of interest. In other words, these sentences contain the meaning of the phenomenon of interest.

Therefore, a balance of research perspectives will be attempted considering both the individual elements and the whole [31] in the context of keeping the enriching notion of the “interpretive circle” active in the analysis [40]. When writing in IPA, the way that ideas are expressed is as important as the ideas themselves because that is what will transmit the meaning of the experiences to the reader who is then inducing their reflection and understanding [32,41]. Themes will be supported in writing the results by displaying participants’ quotes along with a subjective interpretation of their experiences

### Field Notes

Field notes will also be considered throughout the data collection and analysis process. These are mainly to assist in building a description of the context of the interview and analysis processes, which prompt researchers to pay attention to the physical environment and encourage them to reflect and identify any potential bias [42].

### Rigor

In qualitative studies, rigor indicates being precise, meticulous, and firm with accuracy to decrease possible subjectivity [43]. If rigor were lacking, qualitative research would be seen as fictitious and meaningless for enlightening the phenomenon of interest [44].

IPA also aims to safeguard fidelity and integrity despite there being no consensus on specific techniques for establishing rigor in interpretative research [45]. Nonetheless, the idea is that an independent audit of the research methods should still be feasible when defined coherently and where trustworthiness is elegantly attained [45,46].

This study will take into consideration the COnsolidated criteria for REporting Qualitative research (COREQ) guidelines [47]. Among the strategies for ensuring the validity of a study, the concepts of “rich, thick description,” “member checking,” “clarifying research bias,” and “peer debriefing” will be used [24].

### Reflexivity

It is understood that researchers’ behaviors, interests, and knowledge might impact the study atmosphere and data; consequently, critical reflective discerning, or reflexivity, is required during the whole study development [48].

In using van Manen’s framework, controversy arises from the fact that each academic may not comprehend a phenomenon in the same manner [45,49]. This is not difficult to understand since each investigator or academician carries personal preunderstandings, perspectives, and experiences into IPA [49,50]. Thus, the appraisal of researchers’ personal preunderstandings and experiences forms part of reflexivity [49]. The researchers will practice reflexivity during the study.

### Member Checking

Member checking has been defined as criticism attained from interviewees to correct, comment, or approve the investigators’ findings, interpretations, or results [51]. There are many strategies for member checking, such as having a participant review a synthesis of their case report, a copy of the research report, a copy of emerging findings, a complete copy of the transcript, or some combination of these strategies [51]. Automated transcription services will be generated from the audio recordings, and these will be verified and corrected by the authors.

Many scholars doubt that member checking can improve the attributes of qualitative research [47,52]. Nevertheless, the authors will apply member checking because it would be another opportunity to confirm the participants’ interpretations of their perspectives and experiences.

### Triangulation

In IPA, triangulation refers to assessing the value of data through the convergence of discoveries from diverse sources. Triangulation can also refer to analyst triangulation, which is the development of a broader understanding of the phenomenon in question along with improved analysis [53,54].

In this study, such analyst triangulation will be attempted by various methodological perspectives available for observing the data and developing the interview topics followed by the analysis [21,55]. The processes of isolating themes and interpretive analysis, as well as the writing, will be monitored and examined by the authors and research collaborators, hence further fulfilling the praxis of analyst triangulation. More specifically, an experienced qualitative senior researcher (SRB) will revise the work in full at every stage.

## Results

### Participant Sampling

“Purposive sampling” will secure participants with a variety of characteristics in this study [46,55]. To satisfy one of the secondary aims, “criterion sampling” is the type of purposive sampling used [56] to look for at least two participants meeting the criterion of having the experience of living with a maxillary anterior single-implant tooth that clinicians objectively consider esthetically unsatisfactory, but the participants consider satisfactory. Thus, this study attempts to include a minimum of eight patients whether or not saturation had been achieved earlier. Moreover, understanding the satisfaction of an inadvertently homogenous sample of participants, who would have either pleasing or displeasing outcomes based on objective parameters, would unnecessarily limit a diverse interpretation of the study as a whole.

To facilitate identification of diversity among participants relating to the identification of esthetically pleasing and displeasing cases from a clinician perspective, standardized photographs from a digital camera (Canon EOS Rebel T7i; Canon Inc) with a macro lens (EF 24-105 mm f/4L IS II USM; Canon Inc) and maxillary virtual cast models (ie, Standard Tessellation Language [STL] files in Preview app version 11.0; Apple Inc) generated from an intraoral scan (TRIOS intraoral scanner; 3Shape A/S) will be obtained for each participant, based on the materials required for using a validated objective esthetic index (the pink esthetic score [PES]/white esthetic score [WES]) [57-59]. Two experienced clinician researchers (KIA and KI), calibrated for esthetic analyses, will independently evaluate these materials, and the resulting objective scores will be used to categorize the participants descriptively. A score of 6 (out of a maximum of 10) for either PES or WES, and 12 (out of a maximum of 20) for PES and WES combined will generally be considered satisfactory.

The study inclusion criteria and the patients’ characteristics are provided in Table 1. The exclusion criteria are as follows: (1) two or more adjacent maxillary premolar or anterior teeth restored with implants; (2) any missing maxillary premolar or anterior teeth not yet restored with a fixed dental prosthesis; (3) lack of attendance at follow-up appointments regularly scheduled in the Faculty of Dentistry clinics; and (4) inability to converse in English.

Ideally, our sample would also have an heterogenous distribution of age and sex. In other words, at least four men and four older participants would be included. As is the nature of phenomenological research, the sampling strategy is adaptable, depending on the research needs. The in-depth and rich nature of individual cases is another important factor in defining sample size in phenomenological research [35].

**Table 1:** Purposive sampling for the study: participant characteristics.

Variable	Inclusion criteria	Rationale
Sex	Female or male	To include experiences from both sexes
Age	People older than 18 years	At age 19 years, people are considered adults in British Columbia. A person rarely receives an implant prior to 19 years of age
Dental condition	Partially edentulous patients who had a maxillary anterior tooth or premolar replaced by an implant-supported crown, where natural teeth have adjacent and contralateral teeth	To explore the experiences and perspectives of this sample exposed to the homogenous intervention
Esthetic objective score	Acceptable or unacceptable pink esthetic score and white esthetic score	To report a range of thoughts and to explore whether patients’ implant crown experiences are influenced by the objective esthetic outcomes
Treatment stage	After the patient had the final implant crown for at least 12 months	To include all ranges of experiences at different follow-up periods
Capacity to consent	Stable mental health	The participant should be able to give details of the experience during the interview and be able to provide or reject consent
Language	Conversant in English	Because translation will not be considered, the interviews will have to be carried out in English
Location	Regular patient of the University of British Columbia Dental Clinics	This is the location where the treatment has been fully delivered (surgery and prosthesis) and where the study will be conducted

### Data Saturation

The concept of saturation posits that new information does not improve a previous understanding of the studied phenomenon; hence, in IPA, it signifies there is no need to conduct more interviews. Saturation has been criticized for the inherent degree of vagueness and discrepancies in determining how to accomplish, measure, and judge it appropriately. Moreover, it has been stated that when considering the sample size of a qualitative study, six to 10 interviews may suffice to reach data saturation, but only when the research question is considered to be well focused and the participants’ characteristics are not overly diverse [60]. As a reasonable comparison, previous qualitative studies on patients with dental implants included between five and 16 participants [17,61,62]. As noted, this study estimates achieving saturation in the process of including a minimum of eight participants.

### Additional Data Collection

Each participant will be scheduled for a maximum of 2.5 hours for the background data collection and interview session. At the beginning of the session, the consent form will be read and signed. The participants will have two digital photographs taken of the maxillary teeth (a social smile expression [dynamic position] with and without standard dental cheek retractors to display the teeth). A digital impression or intraoral scan of the upper jaw will be performed, and a semistructured interview will be conducted (see the Methods section). A subjective self-administered questionnaire will be applied with the purpose of having repository data in case this may be needed for a quantitative objective that has not been considered in the present protocol. At the end of the session, a payment receipt form will be signed by the participants.

### Ethics

Ethical principles for medical research involving human subjects specified in the Declaration of Helsinki were considered when applying for ethical approval [63]. A Minimal Risk Certificate of Approval has been obtained from the UBC Behavioral Research Ethics (BREB) Board. This warrants due consideration of the participants’ safety, dignity, and well-being, in addition to respecting their rights. Ethical approval was granted by the UBC BREB Board (H19-00107) in May 2019.

### Peer Review

This study has been funded by two American dental foundations. This study protocol has undergone an independent, high-quality, and impartial peer review by its funders and four external reviewers of *JMIR Research Protocols*.

### Dissemination of the Findings

The authors will prepare manuscripts and disseminate the findings through appropriate peer-reviewed journals.

### Availability of Data and Materials

This protocol does not contain data sets. All data generated or analyzed during this study will be included in a data repository in the published article.

## Discussion

### Study Significance

This paper describes how to use quantitative data for further categorizing the recruited participants purposively and how the qualitative data will be synthesized in an interpretive phenomenological approach. This is valuable since a previous study about mandibular implant overdentures suggested that patient satisfaction may be influenced by several factors that may not be considered in previous quantitative studies [6]. To the authors’ knowledge, this is the first study specifying qualitative methods in the field of oral health and dental medicine to explore patient perceptions of experiences with single-tooth implant restoration in the maxillary anterior region of the mouth. This information is expected to deepen dental clinicians’ understanding of the unexplained results previously reported in quantitative studies about this particular group of the dental population. The findings can also inform further development of PROMs to provide clinicians with tools to improve their communication with these patients [64]. This may also direct future developments in dental intervention satisfaction research and the creation of patient information and education resources [65], concentrated on strategic areas to be highlighted after data analysis.

### Limitations

There is an exceptional opportunity to understand holistically (eg, behaviors, perceptions, beliefs, and emotions) the phenomenon in question for the first time by analyzing the rich detailed data to be collected from the in-depth interviews. Quantitative approaches have failed to do so since their settings might not reproduce genuine comportment, and the interpretation of unusual or conflicting outcomes is nearly impossible [21]. However, the authors acknowledge that qualitative research is not a limitation-free approach. The main drawbacks of qualitative methods are the complexities to collect and analyze the data, which consume resources (ie, time and money), and the imperfect ability to envisage and generalize (ie, external validity) the results [21]. Nevertheless, the proposed qualitative study is required to complement what is evidenced by the vast amount of quantitative studies available [4,59,65-67].

### Conclusions

This is the first IPA study protocol to propose exploring dental patients’ lived experience after having a missing maxillary anterior tooth replaced by a single-tooth implant. The study will use a rigorous design and methodology to capture the lived experience of this group of the population. The findings may provide tools to clinicians for improved understanding and communication with dental patients. This qualitative study will recruit participants from only one university clinic and may not be considered entirely representative of all Canadian dental patients with a single-tooth implant in the esthetic zone.

## Appendix

Multimedia Appendix 1 Interview guide about the experiences and perceptions of patients with a single-tooth implant crown in the esthetic zone.

## Abbreviations

BREB: Behavioral Research Ethics Board
IPA: interpretative phenomenological analysis
PES: pink esthetic score
PROM: patient-reported outcome measure
UBC: University of British Columbia
WES: white esthetic score
