# Supplementary material for: Understanding the Lived Experience of North American Dental Patients With a Single-Tooth Implant in the Upper Front Region of the Mouth: Protocol for a Qualitative Study
Source: JMIR Res Protoc. 2021 Jun 18;10(6):e25767. doi: 10.2196/25767 (PMC8277304; doi:10.2196/25767)
Supplement: Multimedia Appendix 1 [file resprot_v10i6e25767_app1.pdf]

APPENDIX A

Interview guide about the experiences and perceptions of patients with a single-tooth implant crown in the esthetic zone

---

Initial greeting: My aim here is to have a conversation with you about your thoughts on the dental implant you have in the upper front of your mouth and how satisfied you may (or may not) be with the outcome.
I am not concerned that you have a constant flow of ideas. Instead, I want to help you think out loud to teach me about your experience with your dental implant.

Part 1: Introductory Background Questions (Icebreaker)

a. How did you come to lose your tooth?

Part 2: Is about your Overall Satisfaction with the Implant-tooth

a. How well has the implant-tooth met our expectations? (is this what you hoped for?)
   - In what ways? (In what ways NOT?)
   - Is there anything else you would like to comment about it?
   - Do you have any other positive thoughts or experiences with your dental-implant?
   - Do you have any other negative thoughts or experiences with your dental-implant?

b. Would you have another dental implant if it was an option in the future?
   - Why or why not?

c. Would you recommend this dental implant treatment to others?
   - Why or why not?

d. Is there anything that could be improved so other patients may have a better outcome?
   - In what ways?

Part 3: Is about your Satisfaction with the Appearance (or Look) of your Implant-tooth

a. How has your implant-tooth affected your satisfaction with appearance or smiling?
   - In what ways? (In what ways NOT?)
   - How are you managing with this? (does this meet your expectations?)
   - How important is this to you?

b. How well does the white ceramic portion of your implant-tooth blend naturally with the other teeth?
   - How important is this to you?
   - Does this aspect have any other meaning for you?

c. How well does the pinkish gum blend naturally surrounding your implant-tooth?
   - How important is this to you?
   - Does this aspect have any other meaning for you?

Part 4: Is about Satisfaction with your Functioning and Social Experiences relating to your Implant-tooth

a. Has your implant-tooth affected your Functioning or Social Experiences in any ways?
- In what ways?
- How are you managing with this? (does this meet your expectations?)
- How important is this to you?
- Does this have any other meaning for you?

b. Specific aspects (Functioning or Social Experiences)
- Chewing? - Choice of food? - Food trapping?
- Taste? - Swallowing or Digestion?
- Speaking or Singing?
- Ability to Clean and How Fresh your Breath feels?
- Any other comments?

Part 5: Any other important experiences that affect satisfaction with your Implant-tooth, such as Complications, Maintenance or Financial Aspects

a. Have you had any Complications or Maintenance difficulties with your implant-tooth?
- When and how did this difficulty start?
- How are you managing with this? (does this meet your expectations?)
- How much does this complication influence (contribute to or diminish) your satisfaction?
- Does this have any other meaning for you?

b. How satisfied are you with your recall dental appointments for your implant-tooth?
- How important is this?
- What can be improved?
- How clearly were maintenance/hygiene instructions given? (communication with the clinical provider)
  - Why or why not?
  - What can be improved?

c. In your opinion, how reasonable were the fees for the dental-implant?
- How important is this?

d. Do you have any other thoughts on your satisfaction with the implant-tooth?

Part 6: Surgical aspects of the Implant-tooth Treatment

a. What was your surgical experience like?

Thank you
